# The Role of Red Meat and Flavonoid Consumption on Cancer Prevention: The Korean Cancer Screening Examination Cohort

**DOI:** 10.3390/nu9090938

**Published:** 2017-08-25

**Authors:** So Young Kim, Gyung-Ah Wie, Yeong-Ah Cho, Hyun-hee Kang, Kyoung-A. Ryu, Min-Kyong Yoo, Shinyoung Jun, Seong-Ah Kim, Kyungho Ha, Jeongseon Kim, Yoon Hee Cho, Sangah Shin, Hyojee Joung

**Affiliations:** 1 Department of Clinical Nutrition, Research Institute & Hospital, National Cancer Center, Goyang 10408, Korea; gawie@ncc.re.kr (G.-A.W.); yacho@ncc.re.kr (Y.-A.C.); saltstars@ncc.re.kr (H.-h.K.); rka2007@ncc.re.kr (K.-A.R.); mkyoo52@ncc.re.kr (M.-K.Y.); 2 Graduate School of Public Health, Seoul National University, Seoul 08826, Korea; tlsdud426@snu.ac.kr (S.J.); ksacute@snu.ac.kr (S.-A.K.); j015kh@snu.ac.kr (K.H.); 3 Molecular Epidemiology Branch, National Cancer Center, Goyang 10408, Korea; jskim@ncc.re.kr; 4 Department of Biomedical and Pharmaceutical Sciences, The University of Montana, Missoula, MT 59812, USA; yoonhee.cho@umontana.edu; 5 Institute of Environmental Medicine, Seoul National University Medical Research Center, Seoul 03080, Korea; ssa8320@snu.ac.kr; 6 Department of Preventive Medicine, Seoul National University College of Medicine, Seoul 03080, Korea; 7 Institute of Health and Environment, Seoul National University, Seoul 08826, Korea

**Keywords:** cancer, red meat, flavonoid, diet, cohort study

## Abstract

Markedly increased red meat consumption is a cancer risk factor, while dietary flavonoids may help prevent the disease. The purpose of this study was to investigate the associations of red meat and flavonoid consumption with cancer risk, based on data from 8024 subjects, drawn from the 2004–2008 Cancer Screening Examination Cohort of the Korean National Cancer Center. Hazard ratios (HRs) were obtained by using a Cox proportional hazard model. During the mean follow-up period of 10.1 years, 443 cases were newly diagnosed with cancer. After adjusting for age, there was a significant correlation between cancer risk and the daily intake of ≥43 g of red meat per day (HR 1.31; 95% CI 1.01, 1.71; *p* = 0.045), and total flavonoid intake tended to decrease cancer risk (HR 0.70; 95% CI 0.49, 0.99; highest vs. lowest quartile; *p*-trend = 0.073) in men. Following multivariable adjustment, there were no statistically significant associations between flavonoid intake and overall cancer risk in individuals with high levels of red meat intake. Men with low daily red meat intake exhibited an inverse association between flavonoid consumption and cancer incidence (HR 0.41; 95% CI 0.21, 0.80; highest vs. lowest; *p*-trend = 0.017). Additional research is necessary to clarify the effects of flavonoid consumption on specific cancer incidence, relative to daily red meat intake.

## 1. Introduction

Cancer is a major, life-threatening global disease, with approximately 14 million new cases and 8 million cancer-related deaths in 2012 [1]. In 2013, the probability of developing cancer in Korea was 36.2% [2]. Since 1983, the number of cancer-related deaths has steadily increased, and according to the 2015 annual cause-of-death report, cancer-related deaths accounted for 27.9% of all deaths [3]. The economic burden of cancer has increased from 11,424 to 20,858 million USD between 2000 and 2010 [4].

According to the World Cancer Research Fund (WCRF) and the American Institute for Cancer Research (AICR), dietary factors including the intake of red meat, sodium, fruits, and vegetables relate to cancer risk [5]. In 2015, the International Agency for Research on Cancer (IARC) classified red and processed meats as “probably carcinogenic to humans” and “carcinogenic to humans”, respectively [6]. Several cohort and case-control studies showed a positive correlation between red meat consumption and colorectal cancer risk [7,8,9,10,11,12,13,14]. Studies of pancreatic cancer [15,16,17,18] and prostate cancer [19,20,21] also showed a positive correlation between red meat consumption and increased cancer incidence. Processed meat consumption positively correlates with gastric cancer [22,23]. The WCRF/AICR recommended limiting red meat consumption to 300 g/week (43 g/day), for cancer prevention [5].

Diets high in fruits and vegetables are likely exert a protective effect on cancer incidence. Epidemiologic studies indicated that diets high in fruits and vegetables are inversely associated with the incidence of several cancers [24,25,26,27]. Flavonoids are a group of bioactive polyphenolic compounds that naturally occur in vegetables, nuts, fruits, and beverages such as coffee, tea, and red wine. The flavonoid content of these foods may partially account for their protective cancer effects [28]. Epidemiologic studies suggested that diets rich in flavonoids are associated with reduced risk of esophageal [29], gastric [29,30], colorectal [31], high-grade prostate [32], and lung cancers [33].

Over recent decades, Korea has experienced rapid economic growth and industrialization. These changes accompanied dietary shifts, from a traditional rice-based diet to a Westernized diet [34]. As an example, in Korea, red meat intake has steadily increased since the 1980s. According to the 2015 Korea National Health and Nutrition Examination Survey, the average Korean consumes approximately 109.4 g of red meat per day, nearly three times higher than rates seen 30 years ago, and 2.5 times higher than levels recommended by the WCRF/AICR. In the meantime, vegetable consumption was relatively unchanged (291.0 g/day in 2015), and fruit consumption increased by 112.6 g/day, over this 30-year period [35].

Only a few case-control studies examine the association between flavonoid intake and cancer risk in Korea [36,37], and there are no Korean cohort studies on the potential effects of interactions between red meat and flavonoid intake on cancer risk. Therefore, the present study sought to investigate the associations between total flavonoid intake and cancer risk relative to dietary red meat consumption.

## 2. Materials and Methods

### 2.1. Study Population

We obtained data from the Cancer Screening Examination Cohort (CSEC) of the National Cancer Center (NCC) in Korea. The subjects who completed a baseline questionnaire, health examination, and nutrition survey were included and those with a previous cancer history, as well as those with an abnormally low, or high, daily energy intake (<700 kcal or >5000 kcal) were excluded. Details regarding the rationale and methods of the CSEC are published elsewhere [38].

All subjects completed a questionnaire about their sociodemographic (sex, age, income, occupation, education, marital status, physical activity, alcohol consumption habits, and smoking habits) and medical histories at the baseline interview. We obtained data pertaining to body mass index (BMI), cancer site, and the time of cancer diagnosis using the Korean NCC electronic medical record (EMR) database. For all subjects, we confirmed cancer incidence after baseline measures, through the Korean Central Cancer Registry, or NCC EMR.

All subjects provided written informed consent, and the Institutional Review Board of the Korean NCC (NCCNCS-09-274) approved this study.

### 2.2. Assessment for Red Meat and Flavonoid Intake

Dietary information was obtained from a 3-day food record. Subjects recorded all foods and drinks consumed over a 3-day period (1 weekend day and 2 weekdays), within a week. To increase the accuracy of the records, registered dietitians (RD) confirmed all written entries during face-to-face interviews, using food models and supplemental instruments. For each subject, we measured daily energy consumption, macronutrients, micronutrients, individual foods, and food groups using the CAN-Pro 3.0 (Korean Nutrition Society, Seoul, Korea).

The WCRF/AICR recommends that red meat consumption be limited to 300 g/week (43 g/day) [5]. We used this recommendation as the criterion for a risk-based analysis of red meat consumption. In the present study, red meat referred to beef, pork, lamb, goat, and processed meats. Processed meats included meats contained in processed foods such as bacon, ham, sausage, and internal organs (offal, such as the brain, liver, heart, intestines, and tongue).

To estimate flavonoids intake, we used a flavonoid database of common Korean foods (KFDB, Seoul, Korea), developed and maintained by Seoul National University. A detailed description of this database is available elsewhere [39]. The KFDB includes seven major flavonoid subclasses and their thirty-one main flavonoid aglycones, including flavonols (quercetin, kaempferol, myricetin, and isorhamnetin), flavones (luteolin and apigenin), flavanones (eriodictyol, hesperetin, and naringenin), flavan-3-ols (catechin, epicatechin, epigallocatechin, theaflavin, theaflavin-3-gallate, theaflavin 3′-gallate, theaflavin 3, 3′-digallate, and thearubigin), anthocyanidins (cyanidin, delphinidin, malvidin, pelargonidin, peonidin, and petunidin), isoflavones (daidzein, genistein, and glycetin), and proanthocyanidins (dimers, trimers, 4–6 monomers, 7–10 monomers and >10 monomers). We linked food consumption data from the 3-day food record with the KFDB to estimate flavonoid intake. We expressed flavonoid content as aglycones (mg/day), and calculated the flavonoid intake from individual food items by multiplying the flavonoid content by the total amount of food (in grams) consumed. Daily total flavonoid intake was the daily mean of the summation of individual flavonoid intake from all food sources reported within the 3-day food record.

### 2.3. Statistical Analysis

General characteristics of subjects are displayed as the percentages, means, and quartiles, and compared using a chi-square test for categorical variables and *t*-tests and *p* trend test, for continuous variables.

Total flavonoid and red meat intakes were categorized using cohort-wide quartiles or WCRF recommendations, adjusted for total energy intake using the residual method [40]. We calculated hazard ratios (HRs) and 95% confidence intervals (CIs) using the Cox proportional hazards regression model, adjusting for covariates. Potential confounding variables were identified using a self-reported questionnaire. Continuous variables considered as confounders included age (years), energy intake (kcal/day), and BMI (kg/m^2^), whereas categorical variables considered as confounders included sex (women, men), physical activity (yes, no), alcohol consumption (yes, no), smoking (yes, no), income (<4,000,000 KRW (₩)/month, 4,000,000–7,000,000 KRW (₩)/month, and ≥7,000,000 KRW (₩)/month), and marital status (unmarried, married, and divorced or widowed).

HRs for flavonoid intake in subgroups were analyzed as follows: red meat intake; sex (women, men); cancer sites (gastrointestinal (GI) cancer, non-gastrointestinal (non-GI) cancer); and, age (<50 years, ≥50 years). To test for linear trends across quartiles of total flavonoid intake, we created a continuous variable using the median value of each quartile of total flavonoid intake.

We used the STATA program, Version 14.0 (STATA Corporation, College station, TX, USA), for all statistical analyses, and a two-sided *p* value < 0.05 was considered statistically significant.

## 3. Results

A total of 26,815 subjects, aged 20 years and older, participated in the CSEC between September 2004 and December 2008. The 8179 subjects who completed a baseline questionnaire, health examination, and nutrition survey were initially included in our study pool. Following the exclusions for prior cancer history (*n* = 79) and those with abnormally high or low, daily energy intake (<700 kcal or >5000 kcal, *n* = 29), 8024 subjects remained and were included in the final analyses.

The median follow-up was 10.1 years (total 80,927.0 person-years). According to the International Classification of Diseases (ICD)-10 Codes C00-C99 [41], 443 new cancer cases, 148 cases of GI cancer and 295 cases of non-GI cancer, were identified during the follow-up period, ending 7 April 2017. GI cancers included cancer of the esophagus, stomach, gallbladder, pancreas, liver, colon, rectum, and anus.

The baseline characteristics of the study population are displayed in Table 1. Men and women who consumed at least 43 g of red meat per day tend to be younger, and consumed fewer fruits, vegetables, and flavonoids. Men and women with a high total flavonoid intake consumed more fruits and vegetables, and less red meat, as compared to those with a lower total flavonoid intake. These subjects also reported more frequent physical activity.

Cancer-related HRs for red meat and flavonoid intake are presented in Table 2. After age adjustment, the overall cancer risk in men was significantly increased for those who consumed more than 43 g of red meat per day (or 300 g/week), as compared to those who ate less than 43 g/day (HR 1.31; 95% CI 1.01, 1.71; *p* = 0.045); however, this significant association was weakened after multivariable adjustment (HR 1.21; 95% CI 0.91, 1.61; *p* = 0.180). In men younger than age 50, there was a significant correlation between red meat intake, of at least 43 g/day, and cancer incidence after multivariable adjustment (HR 2.03; 95% CI 1.14, 3.64; *p* = 0.017) (data not shown). For non-GI cancers, the HR of men who consumed more than 43 g of red meat per day was significantly higher than that observed in men who consumed less than 43 g/day, after age adjustment (HR 1.45; 95% CI 1.03, 2.06; *p* = 0.034). We observed no similar association for GI cancers. In women, red meat consumption did not affect cancer risk.

Table 2 displays the relationships between flavonoid consumption and cancer risk. The total flavonoid intake tended to decrease overall cancer risk in men, after age adjustment (HR 0.70; 95% CI 0.49, 0.99; highest vs. lowest quartile; *p*-trend = 0.073); however, this trend was weakened after multivariable adjustment (HR 0.69; 95% CI 0.47, 1.00; highest vs. lowest quartile; *p*-trend = 0.075). In men over 50 years of age, those in the highest flavonoid intake quartile had a significantly lower incidence of overall cancer, as compared to men in the lowest quartile after multivariable adjustment (HR 0.62; 95% CI 0.38, 0.99; highest vs. lowest quartile; *p*-trend = 0.054) (data not shown). In cancer subgroup analysis, flavonoid intake did not affect GI and non-GI cancer risk in either men or women.

We stratified our analysis of flavonoid intake and cancer incidence by the amount of red meat consumed per day and sex (Table 3). After multivariable adjustment, there were no statistically significant associations between flavonoid consumption and cancer risk in subjects who consumed more than 43 g of red meat per day. However, in men who consumed less than 43 g of red meat per day, those in the highest quartile of flavonoid intake had a significantly lower incidence of overall cancer, as compared to men in the lowest quartile after multivariable adjustment (HR 0.41; 95% CI 0.21, 0.80; highest vs. lowest quartile; *p*-trend = 0.017).

## 4. Discussion

This study is the first prospective cohort study to examine the cancer-protective effect of dietary flavonoids in Koreans with high levels of daily red meat consumption. Higher intakes of red meat tended to increase cancer risk, and higher dietary flavonoid intake tended to reduce cancer risk. However, flavonoid intake did not reduce cancer risk in those who exhibited high levels of red meat consumption.

In 2015, IARC issued a press release regarding the carcinogenicity of red and processed meat consumption [6]. Recent meta-analyses reported that red meat consumption is a risk factor for cancer at several sites [16,17,23,42,43,44,45,46]. Our study showed similar results. In men, high levels of red meat consumption increased cancer risk, but after multivariate adjustment, this association was weakened. However, there was no significant association between the amount of red meat consumed and cancer incidence in women. Similar to that observed in previous studies, we found contradictory results for the association between red meat consumption and cancer incidence in women. Recent meta-analyses have shown that red meat consumption increases the incidence of pancreatic cancer in men but not in women [16,17]. In those studies, the authors explained that the lower absolute intake of meat and meat-derived carcinogens in women than in men, and men preferring to consume fried, barbecued, or grilled meat more than women may contribute to the different results according to sex. In the present study, we did not consider the cooking method in the analysis. Previous studies have also suggested that if there is a threshold effect with the increased risk for pancreatic cancer only at very high levels of red meat consumption, a positive association may be more likely to be observed in men. In our study, it is possible that red meat intake increased cancer risk only in men, because the red meat intake was significantly higher in men than in women (85.3 g/day vs. 59.5 g/day, respectively). In addition, the results may be attributable to the difference in the ratio of red meat-to-antioxidant (such as flavonoids) intake. In contrast to red meat consumption, fruit and vegetable consumption was significantly higher in women than in men (418.0 g/day vs. 405.6 g/day, respectively), as well as total flavonoid consumption (150.6 mg/day vs. 133.0 mg/day, respectively). However, the effect of red meat on cancer incidence may differ by cancer type. In a Japanese study, in women, the risk for proximal colon cancer was found to increase with high red meat intake, but the risk for distal colon cancer did not [12]. However, in a Swedish study, red meat consumption was associated with increased distal colon cancer incidence, but not proximal cancer incidence among women [8]. We have not been able to analyze the incidence by cancer types owing to insufficient data. Therefore, additional study is necessary considering the effects of the interactions of dietary factors on the cancer risk by cancer site and sex.

In Korea, there are rapid shifts towards a Westernized diet recently, characterized by a high consumption of meat and animal products. These shifts are particularly strong in younger populations [47]. In the present study, high red meat intake increased cancer risk, particularly for men younger than 50 years of age. Men under 50 years of age had a significantly higher red meat intake than men over 50 years of age (82.2 g/day vs. 65.6 g/day, respectively). In addition, the proportion of people who consume more than the recommended intake proposed by the WCRF/AICR was also higher in men under 50 years of age than in men over 50 years of age (71.4% vs. 58.5%, respectively). Additional research to explore dietary factors related to cancer prevention in younger adults is required.

Men within the highest flavonoid intake quartile marginally decreased their incidence of cancer, as compared to those in the lowest quartile, after multivariate adjustment (HR 0.69, 95% CI 0.47, 1.00; highest vs. lowest quartile; *p*-trend = 0.075). For men over 50 years of age, the highest quartile had a significantly lower cancer risk than the lowest quartile after multivariable adjustment (HR 0.62, 95% CI 0.38, 0.99; *p*-trend = 0.054) (data not shown). Several recent studies have shown that flavonoid intake reduces the risk of esophageal [48], lung [33], prostate [32], pancreatic [49], gastric [29], colorectal [31], and bladder cancer [50]. Other studies showed no association between flavonoid intake and cancer incidence [51,52,53]. To our knowledge, there are few prospective studies on the relationship between flavonoid and cancer risk in Koreans, and only a few case-control studies investigating the relationship between flavonoid intake and cancer incidence [36,37]. In one of these studies, flavonoid intake and gastric cancer incidence were significantly correlated, particularly among Korean women [36]. Fink et al. reported that dietary flavonoid intake was inversely correlated with breast cancer incidence in post-menopausal women, and the relationship was not observed in pre-menopausal women [54]. Although menopause-related hormonal changes are an important factor in studies of older women, we could not consider these changes in our analysis.

In the present study, high flavonoid intake did not mitigate cancer risk in those with high red meat consumption. Cancer risk decreased with increased flavonoid intake only for men who consumed less than 43 g of red meat per day. In this study, mean daily total flavonoid intake was 140.9 mg, which was lower when compared with the means of previous studies [39,50]. The lower total flavonoid intake, found among subjects in this study, may attenuate dietary flavonoid effects on cancer risk in men who consume high amounts of red meat. On the other hand, those who consumed more red meat intake had lower flavonoid intake than those who did not consume large amounts of red meat. This may have affected outcomes observed in subjects with high red meat intake. A recent study reported that high intake of red meat was found to be associated with a higher risk for all-cause and cardiovascular disease mortality, and the increased risks were consistently observed, even with high intake of fruits and vegetables [55]. These results suggest that mortality related to high red meat intake could not be counterbalanced by the consumption of preventive foods such as fruits and vegetables. This also suggested that increased cancer risk due to high red meat consumption could not be counterbalanced by an increase in flavonoid intake.

This study had the advantages of being a large prospective cohort study, investigating the associations between diet and cancer risk in Korean subjects for the first time. All data for red meat and flavonoid intakes were obtained from 3-day dietary records, which can accurately measure subjects’ usual dietary intake while minimizing recall bias and reverse causation.

On the other hand, our study had a number of limitations. First, our mean follow-up period was 10.1 years, which may not be enough to identify sufficient number of incident cancer cases for analysis. Second, subjects were self-motivated and recruited from hospital settings. Consequently, selection bias cannot be completely discounted. Third, flavonoid intake might be underestimated because the coverage of the KFDB for common Korean foods are 49% of food items and 76% of food intake, and that from dietary supplements were not estimated in our analysis. Finally, it is difficult to elucidate the overall effects of dietary flavonoids and other phytochemicals with anticancer properties and interactions with other known dietary risk factors such as red meat. Further research with comprehensive dietary exposure analysis should be followed.

In summary, the results showed that red meat intake increased cancer risk; while flavonoid intake decreased cancer risk, and increased cancer risk from high red meat consumption could not be counterbalanced by consuming flavonoid. Further research on the effect of interaction among dietary factors on specific site cancer is necessary among Koreans.

## Figures and Tables

**Table 1:** General characteristics of the study participants by the levels of red meat and flavonoid intake.

	Red Meat Intake			Flavonoid Intake (Quartile)				
	<43 g/Day	≥43 g/Day	*p*-Value ^1^	Q1	Q2	Q3	Q4	*p*-Value ^1^
Men (*n* = 4402)								
Age (years)	51.2 ± 9.1 ^2^	48.3 ± 9.3	<0.0001	47.2 ± 9.1	48.8 ± 9.4	50.1 ± 9.3	51.1 ±9.1	<0.0001
Income (≥4,000,000 KRW, %)	55.4	62.2	<0.0001	55.4	60.5	59.7	64.0	0.015
Marital status (married, %)	93.9	93.0	0.081	92.2	93.5	93.2	94.4	0.037
Smoking (yes, %)	73.4	78.9	<0.0001	81.1	79.2	75.7	72.1	<0.0001
Alcohol drinking (yes, %)	85.6	86.5	0.268	85.6	86.7	86.4	86.0	0.192
Regular physical activity (yes, %)	45.6	48.8	0.150	44.0	45.7	49.0	52.1	<0.0001
Dietary intake from 3-day records								
Energy intake (kcal/day)	2026.8 ± 474.0	2060.8 ± 457.2	0.0207	2024.9 ± 493.1	2051.1 ± 441.4	2085.6 ± 463.1	2034.6 ± 452.3	<0.0001
Red meat intake (g/day)	25.0 ± 18.2	117.1 ± 63.7	<0.0001	95.6 ± 75.8	89.4 ± 67.5	84.1 ± 68.2	72.0 ± 59.3	<0.0001
Fruits and vegetables (g/day)	434.4 ± 293.8	390.4 ± 242.6	<0.0001	261.2 ± 193.4	357.3 ± 207.3	436.0 ± 225.1	567.7 ± 306.2	<0.0001
Dietary flavonoids intake (mg/day) ^3^	145.9 ± 133.2	126.2 ± 109.3	<0.0001	44.5 ± 13.1	82.8 ± 11.8	132.4 ± 17.5	272.4 ± 160.5	<0.0001
BMI (kg/m^2^)	24.6 ± 2.6	24.8 ±2.7	0.0161	24.7 ± 2.8	24.8 ± 2.7	24.7 ± 2.6	24.7 ± 2.6	-
Women (*n* = 3622)								
Age (years)	50.0 ± 9.0	46.4 ± 8.9	<0.0001	46.2 ± 9.1	47.7 ± 9.3	48.3 ± 9.0	49.2 ± 9.0	<0.0001
Income (≥4,000,000 KRW, %)	46.3	51.8	0.002	50.0	50.4	50.1	48.3	0.892
Marital status (married, %)	83.5	87.5	<0.0001	84.9	88.2	86.2	84.5	0.044
Smoking (yes, %)	5.5	7.8	0.045	8.9	7.4	5.6	5.6	0.102
Alcohol drinking (yes, %)	42.6	51.2	< 0.0001	50.0	50.5	45.6	47.4	0.261
Regular physical activity (yes, %)	34.3	34.5	0.609	27.8	33.7	35.1	41.0	<0.0001
Dietary intake from 3-day records								
Energy intake (kcal/day)	1632.2 ± 418.3	1619.4 ± 396.7	0.3537	1581.6 ± 405.5	1629.9 ± 411.7	1657.9 ± 408.8	1629.0 ± 391.5	<0.0001
Red meat intake (g/day)	16.7 ± 14.1	86.8 ± 52.8	<0.0001	65.0 ± 57.7	60.8 ± 53.9	57.6 ± 52.4	54.6 ± 52.5	<0.0001
Fruits and vegetables (g/day)	448.5 ± 276.3	398.6 ± 237.5	<0.0001	276.6 ± 195.4	376.6 ± 118.6	453.6 ± 219.9	565.5 ± 283.0	<0.0001
Dietary flavonoids intake (mg/day) ^3^	161.6 ± 136.7	143.6 ± 119.3	<0.0001	49.8 ± 15.9	95.5 ± 13.9	152.8 ± 19.8	304.4 ± 162.8	<0.0001
BMI (kg/m^2^)	23.1 ± 2.9	22.7 ± 3.0	0.0003	22.7 ± 3.1	22.9 ± 3.0	22.9 ± 2.9	22.9 ± 2.9	-

Abbreviations: BMI, body mass index. ^1^ The *t*-test and the trend test were used for continuous variables, and the chi-square test was used for categorical variables. *p* for trends were derived from a generalized linear regression analysis for continuous variables. ^2^ Mean ± SD. ^3^ Energy-adjusted by using the residual method.

**Table 2:** Hazard ratios (HRs) and 95% confidence intervals (95% CIs) for cancer by the levels of red meat and flavonoid intake.

	Total Cancers (*n* = 443)					GI Cancer (*n* = 148)					Non-GI Cancer (*n* = 295)				
	No. of Cases/Person-Years	Model 1 ^1^		Model 2 ^2^		No. of Cases/Person-Years	Model 1 ^1^		Model 2 ^2^		No. of Cases/Person-Years	Model 1 ^1^		Model 2 ^2^	
		HR	95% CI	HR	95% CI		HR	95% CI	HR	95% CI		HR	95% CI	HR	95% CI
Red meats (g/day)															
Men															
<43	82/15,333	1.00		1.00		36/12,672	1.00		1.00		46/13,546	1.00		1.00	
≥43	175/28,932	1.31	(1.01, 1.71)	1.21	(0.91, 1.61)	65/26,213	1.14	(0.76, 1.76)	1.00	(0.64, 1.55)	110/25,595	1.45	(1.03, 2.06)	1.40	(0.96, 2.03)
*p* for trend		0.045		0.180			0.495		0.993			0.034		0.077	
Women															
<43	77/14,320	1.00		1.00		23/11,394	1.00		1.00		54/12,692	1.00		1.00	
≥43	109/22,342	1.00	(0.74, 1.34)	0.84	(0.59, 1.22)	24/20,883	0.89	(0.50, 1.59)	0.79	(0.37, 1.71)	85/19,834	1.03	(0.73, 1.46)	0.85	(0.56, 1.29)
*p* for trend		0.977		0.367			0.702		0.557			0.857		0.440	
Flavonoid intake quartile															
Men															
Q1	70/10,885	1.00		1.00		24/10,841	1.00		1.00		46/9590	1.00		1.00	
Q2	61/11,041	0.79	(0.56, 1.11)	0.81	(0.56, 1.18)	28/10,849	1.06	(0.61, 1.83)	1.22	(0.67, 2.21)	33/9785	0.65	(0.42, 1.02)	0.63	(0.39, 1.02)
Q3	65/11,088	0.78	(0.56, 1.10)	0.73	(0.50, 1.06)	27/10,955	0.93	(0.54, 1.62)	0.89	(0.47, 1.66)	38/9838	0.70	(0.46, 1.08)	0.65	(0.41, 1.03)
Q4	61/11,251	0.70	(0.49, 0.99)	0.69	(0.47, 1.00)	22/11,111	0.72	(0.40, 1.28)	0.76	(0.40, 1.46)	39/9929	0.69	(0.45, 1.06)	0.66	(0.41, 1.06)
*p* for trend		0.073		0.075			0.174		0.208			0.229		0.199	
Women															
Q1	43/9099	1.00		1.00		13/8962	1.00		1.00		31/8066	1.00		1.00	
Q2	45/9090	1.00	(0.66, 1.53)	0.96	(0.58, 1.59)	12/9025	0.79	(0.36, 1.74)	0.69	(0.25, 1.89)	32/8071	1.02	(0.62, 1.68)	1.08	(0.60, 1.94)
Q3	53/9187	1.16	(0.77, 1.74)	0.94	(0.57, 1.55)	12/9160	0.76	(0.34, 1.67)	0.60	(0.21, 1.71)	41/8159	1.30	(0.81, 2.07)	1.09	(0.61, 1.96)
Q4	45/9287	0.96	(0.63, 1.46)	0.91	(0.55, 1.53)	10/9169	0.60	(0.26, 1.38)	0.41	(0.14, 1.26)	35/8223	1.10	(0.67, 1.78)	1.17	(0.65, 2.11)
*p* for trend		0.884		0.727			0.254		0.128			0.631		0.619	

Abbreviations: CI, confidence interval; HR, hazards ratio; GI, gastrointestinal. ^1^ Estimated using the Cox proportional hazards regression model, adjusted for age and sex. ^2^ Estimated using the Cox proportional hazards regression model, adjusted for age, sex, energy intake, BMI, physical activity (yes or no), smoking (yes or no), alcohol use (yes or no), income (<4,000,000 KRW (₩)/month, 4,000,000–7,000,000 KRW (₩)/month and ≥7,000,000 KRW (₩)/month), and marital status (unmarried, married, and divorced or widowed).

**Table 3:** Hazard ratios (HRs) and 95% confidence intervals (95% CIs) for cancer by the level of flavonoid intake at each red meat intake level.

	Total Cancers (*n* = 443)					GI Cancer (*n* = 148)					Non-GI Cancer (*n* = 295)				
Flavonoid Quartile	No. of Cases/Person-Years	Model 1 ^1^		Model 2 ^2^		No. of Cases/Person-Years	Model 1 ^1^		Model 2 ^2^		No. of Cases/Person-Years	Model 1 ^1^		Model 2 ^2^	
		HR	95% CI	HR	95% CI		HR	95% CI	HR	95% CI		HR	95% CI	HR	95% CI
Red meat < 43 g															
Men															
Q1	28/3732	1.00		1.00		11/3761	1.00		1.00		17/3300	1.00		1.00	
Q2	16/3858	0.55	(0.30, 1.02)	0.52	(0.28, 0.99)	10/3778	0.90	(0.38, 2.12)	0.79	(0.33, 1.93)	6/3412	0.34	(0.13, 0.87)	0.36	(0.14, 0.91)
Q3	22/3821	0.70	(0.40, 1.22)	0.54	(0.30, 0.99)	9/3816	0.73	(0.30, 1.77)	0.50	(0.19, 1.30)	13/3388	0.68	(0.33, 1.41)	0.58	(0.27, 1.26)
Q4	16/3922	0.48	(0.26, 0.89)	0.41	(0.21, 0.80)	6/3860	0.47	(0.17, 1.27)	0.43	(0.16, 1.19)	10/3446	0.50	(0.23, 1.10)	0.41	(0.17, 0.97)
*p* for trend		0.051		0.017			0.116		0.080			0.239		0.105	
Women															
Q1	15/3590	1.00		1.00		3/3546	1.00		1.00		12/3156	1.00		1.00	
Q2	18/3548	1.13	(0.57, 2.25)	1.12	(0.51, 2.49)	8/3507	2.18	(0.58, 8.29)	1.83	(0.32, 10.30)	10/3154	0.82	(0.35, 1.91)	0.97	(0.39, 2.42)
Q3	28/3516	1.75	(0.93, 3.30)	1.62	(0.77, 3.42)	7/3556	1.89	(0.48, 7.34)	1.76	(0.31, 9.89)	22/3131	1.82	(0.90, 3.70)	1.66	(0.72, 3.80)
Q4	16/3666	0.99	(0.49, 2.01)	0.84	(0.35, 2.04)	5/3597	1.39	(0.33, 5.83)	1.22	(0.20, 7.68)	10/3251	0.81	(0.35, 1.89)	0.76	(0.27, 2.13)
*p* for trend		0.993		0.764			0.960		0.942			0.907		0.776	
Red meat ≥ 43 g															
Men															
Q1	43/7168	1.00		1.00		15/7083	1.00		1.00		28/6319	1.00		1.00	
Q2	38/7234	0.77	(0.50, 1.21)	0.77	(0.48, 1.25)	14/7118	0.80	(0.38, 1.66)	0.95	(0.42, 2.17)	24/6391	0.76	(0.44, 1.31)	0.68	(0.38, 1.25)
Q3	47/7233	0.91	(0.60, 1.37)	0.93	(0.59, 1.46)	19/7114	1.01	(0.51, 2.01)	1.11	(0.50, 2.44)	28/6418	0.84	(0.50, 1.43)	0.83	(0.47, 1.47)
Q4	47/7297	0.87	(0.57, 1.32)	0.93	(0.58, 1.47)	17/7228	0.85	(0.42, 1.72)	1.02	(0.46, 2.30)	30/6466	0.87	(0.51, 1.46)	0.87	(0.50, 1.54)
*p* for trend		0.779		0.928			0.810		0.902			0.844		0.983	
Women															
Q1	28/5533	1.00		1.00		10/5436	1.00		1.00		18/4939	1.00		1.00	
Q2	26/5534	0.91	(0.53, 1.55)	0.82	(0.43, 1.58)	4/5495	0.36	(0.11, 1.16)	0.38	(0.09, 1.50)	22/4891	1.22	(0.65, 2.28)	1.11	(0.51, 2.41)
Q3	23/5666	0.80	(0.46, 1.38)	0.48	(0.23, 1.04)	4/5608	0.36	(0.11, 1.16)	0.13	(0.02, 1.08)	19/5024	1.04	(0.55, 1.99)	0.71	(0.30, 1.68)
Q4	32/5609	1.01	(0.64, 1.77)	1.08	(0.58, 2.01)	6/5571	0.47	(0.17, 1.31)	0.33	(0.08, 1.30)	26/4980	1.41	(0.77, 2.58)	1.59	(0.77, 3.31)
*p* for trend		0.730		0.705			0.264		0.130			0.314		0.197	

Abbreviations: CI, confidence interval; HR, hazards ratio; GI, gastrointestinal. ^1^ Estimated using the Cox proportional hazards regression model, adjusted for age and sex. ^2^ Estimated using the Cox proportional hazards regression model, adjusted for age, sex, energy intake, BMI, physical activity (yes or no), smoking (yes or no), alcohol use (yes or no), income (<4,000,000 KRW (₩)/month, 4,000,000–7,000,000 KRW (₩)/month and ≥7,000,000 KRW (₩)/month), and marital status (unmarried, married, and divorced or widowed).
