# Erratum: The Role of Red Meat and Flavonoid Consumption on Cancer Prevention: The Korean Cancer Screening Examination Cohort; *Nutrients* 2017, *9*, 938

**DOI:** 10.3390/nu9111174

**Published:** 2017-10-27

**Authors:** So Young Kim, Gyung-Ah Wie, Yeong-Ah Cho, Hyun-hee Kang, Kyoung-A. Ryu, Min-Kyong Yoo, Shinyoung Jun, Seong-Ah Kim, Kyungho Ha, Jeongseon Kim, Yoon Hee Cho, Sangah Shin, Hyojee Joung

**Affiliations:** 1 Department of Clinical Nutrition, Research Institute & Hospital, National Cancer Center, Goyang 10408, Korea; drewpia@ncc.re.kr (S.Y.K.); gawie@ncc.re.kr (G.-A.W.); yacho@ncc.re.kr (Y.-A.C.); saltstars@ncc.re.kr (H.-h.K.); rka2007@ncc.re.kr (K.-A.R.); mkyoo52@ncc.re.kr (M.-K.Y.); 2 Graduate School of Public Health, Seoul National University, Seoul 08826, Korea; tlsdud426@snu.ac.kr (S.J.); ksacute@snu.ac.kr (S.-A.K.); j015kh@snu.ac.kr (K.H.); 3 Molecular Epidemiology Branch, National Cancer Center, Goyang 10408, Korea; jskim@ncc.re.kr; 4 Department of Biomedical and Pharmaceutical Sciences, The University of Montana, Missoula, MT 59812, USA; yoonhee.cho@umontana.edu; 5 Institute of Environmental Medicine, Seoul National University Medical Research Center, Seoul 03080, Korea; ssa8320@snu.ac.kr; 6 Department of Preventive Medicine, Seoul National University College of Medicine, Seoul 03080, Korea; 7 Institute of Health and Environment, Seoul National University, Seoul 08826, Korea

The authors have requested the following corrections to their paper [1].

“So Young Kim ^1,^*” has been changed to “So Young Kim ^1^”. So Young Kim is not the corresponding author.

The email address of So Young Kim has been added to the Affiliation 1: Department of Clinical Nutrition, Research Institute & Hospital, National Cancer Center, Goyang 10408, Korea; drewpia@ncc.re.kr (S.Y.K.); gawie@ncc.re.kr (G.-A.W.); yacho@ncc.re.kr (Y.-A.C.); saltstars@ncc.re.kr (H.-h.K.); rka2007@ncc.re.kr (K.-A.R.); mkyoo52@ncc.re.kr (M.-K.Y.)

The Correspondence section has been changed to “Correspondence: hjjoung@snu.ac.kr; Tel.: +82-2-880-2716”.

The authors apologize for any inconvenience caused to the readers by the change. The change does not affect the scientific results. The manuscript will be updated and the original will remain online on the article webpage.
